# Inoculation with the endophyte *Piriformospora indica* significantly affects mechanisms involved in osmotic stress in rice

**DOI:** 10.1186/s12284-018-0226-1

**Published:** 2018-05-24

**Authors:** Muhammad Abu Bakar Saddique, Zulfiqar Ali, Abdus Salam Khan, Iqrar Ahmad Rana, Imran Haider Shamsi

**Affiliations:** 1 Department of Plant Breeding and Genetics, University of Agriculture, 38040, Faisalabad, Pakistan; 2 Centre of Agricultural Biochemistry and Biotechnology, University of Agriculture, 38040, Faisalabad, Pakistan; 3 Department of Agronomy, College of Agriculture and Biotechnology, Zhejiang University, 310058, Hangzhou, People’s Republic of China; 4 Department of Plant Breeding and Genetics, Muhammad Nawaz Shareef University of Agriculture, 60000, Multan, Pakistan

**Keywords:** *Oryza sativa*, *Piriformospora indica*, Symbiosis, Osmotic stress, Fv/Fm, Phosphorus, Zinc, Proline, Total antioxidant capacity, Pyrroline-5-carboxylate synthase

## Abstract

**Background:**

Rice is a drought susceptible crop. A symbiotic association between rice and mycorrhizal fungi could effectively protect the plant against sudden or frequent episodes of drought. Due to its extensive network of hyphae, the endophyte is able to deeply explore the soil and transfer water and minerals to the plant, some of them playing an important role in mitigating the effects of drought stress. Moreover, the endophyte could modify the expression of drought responsive genes and regulate antioxidants.

**Results:**

Three rice genotypes, WC-297 (drought tolerant), Caawa (moderately drought tolerant) and IR-64 (drought susceptible) were inoculated with *Piriformospora indica* (*P. indica*)*,* a dynamic endophyte. After 20 days of co-cultivation with the fungus, rice seedlings were subjected to 15% polyethylene glycol-6000 induced osmotic stress. *P. indica* improved the growth of rice seedlings. It alleviated the destructive effects of the applied osmotic stress. This symbiotic association increased seedling biomass, the uptake of phosphorus and zinc, which are functional elements for rice growth under drought stress. It boosted the chlorophyll fluorescence, increased the production of proline and improved the total antioxidant capacity in leaves. The association with the endophyte also up regulated the activity of the Pyrroline-5-carboxylate synthase (*P5CS*), which is critical for the synthesis of proline.

**Conclusion:**

A mycorrhizal association between *P. indica* and rice seedlings provided a multifaceted protection to rice plants under osmotic stress (− 0.295 MPa).

**Electronic supplementary material:**

The online version of this article (10.1186/s12284-018-0226-1) contains supplementary material, which is available to authorized users.

## Background

Rice is staple food for nearly half of the world’s seven billion people. Almost 754.6 million tons of paddy are annually produced (FAO 2017). Considering the increasing population and the associated demand of rice there is a need to increase this crop’s yield. Because of its semiaquatic ancestors, rice is very susceptible to drought stress, which drastically affects its growth and grain yield (Yue et al. 2006; Fahad et al. 2017). Drought is one of the major cause of yield reduction in rice belts of different countries (FAO 2017). Drought interferes with and damages morphological, physiological and molecular features that are responsible for growth and development (Farooq et al. 2009a). In the case of rice, low water availability reduces germination, plant biomass, number of tillers, plant height and modifies root angle (Ji et al. 2012; Sokoto and Muhammad 2014; Uga et al. 2015). It decreases transpiration, stomatal conductance, water use efficiency, relative water content, chlorophyll content, photosynthesis, photosystem II activity and affects membrane stability and abscisic acid content (Ding et al. 2014; Yang et al. 2014). Drought induces the accumulation of osmoprotectants like proline, sugars, polyamines and antioxidants (Li et al. 2011; Fahramand et al. 2014) as well as changes in the expression of genes which encode transcription factors and defense related proteins (Nakashima et al. 2014).

Various strategies can help maintaining rice grain yield under drought prone conditions like, exploitation of diversified germplasm (Liu et al. 2015), effective management practices (Haefele et al. 2016) and by exploiting the association of rice with endophytes (Gill et al. 2016). Some progress has been obtained by genetically improving drought tolerance in rice (Serraj et al. 2011). The management of water during rice field preparation and throughout complete crop growth period can help to save water and make it available during drought episodes (Haefele et al. 2016). Moreover, seed priming, foliar application of growth regulators as well as osmoprotectants and the proper application of phosphorus (P), zinc (Zn) and silicon (Si) can mitigate drought effects under limited water environment (Hattori et al. 2005; Kaya et al. 2006; Taiz and Zeiger 2006; Ashraf and Foolad 2007; Tariq et al. 2017; Gill and Tuteja 2010). Finally, symbiotic association with endophytes can help maintain grain production under stress conditions. Hyphae of the mycorrhizal fungi are able to deeply explore the rhizosphere and transport water and minerals to plant roots and keep roots moist even when there is less water availability (Varma et al. 2013).

Among many endophytes, *P. indica* forms symbiotic association with almost every cultivable crop. It extends its mutualistic link with pteridophytes, bryophytes, angiosperms and gymnosperms (Varma et al. 2001). *P. indica* was found to have a positive effect on host plants growth under saline environment, water stress, temperature shocks as well as biotic stresses. Under osmotic stresses, *P. indica* increases the cellular osmolarity of plant and maintains turgor (Gill et al. 2016). Along with many drought responsive genes *P. indica* up regulates an important proline synthesizing gene, *P5CS* (Abo-Doma et al. 2016). Proline is highly soluble and zwitterionic in nature so its higher accumulation do not damage plants. Instead, it stabilizes cellular structures, acts as water substitute through hydrophilic interactions and hydrogen bonding. It also induces drought tolerance by scavenging ROS and by being utilized as energy source after the release of stress (Verslues and Sharma 2010). Under abiotic stress *P. indica* was also found to stabilize chlorophyll in rice leaves (Abadi and Sepehri 2016). By consequence, it increases the maximum quantum yield of PSII (Fv/Fm) (Vahabi et al. 2016; Shahabivand et al. 2017).

Finally, *P. indica* enhances the uptake of P and Zn. High P uptake is responsible for maintaining optimum leaf relative water contents, efficiency of photosystem II and net photosynthetic rate. High phosphorus level decreases malondialdehyde content and increases osmolytes and nitrogenous compounds concentration (Tariq et al. 2017). Zinc is part of antioxidant complexes (Cu/Zn-SOD) and is also important for scavenging ROS that ultimately reduces the damage to cellular membranes (Gill and Tuteja 2010; Ngwene et al. 2016). In response to these valuable services, the plant could devote up to 15–20% of the produced photosynthates to this fungus but the extent of benefits generally offsets this cost (Varma et al. 2001). In the present study, rice plants have been inoculated with *P. indica* under polyethylene glycol-6000 (PEG-6000) induced water stress in hydroponic conditions. The effects of the symbiotic association under osmotic stress were analyzed on seedling biomass, root and shoot length, P and Zn uptake, expression of *P5CS* gene, integrity of grana in chloroplasts, level of Fv/Fm, proline content and total antioxidant capacity (TAC). Whereas, in most of the previous research articles, the interaction between rice roots and *P. indica* had been studied under salt and heavy metal stress. It had been reported that this symbiotic fungus enhances the seedling biomass, length of root and shoot, chlorophyll content and proline concentration under salt stress (Jogawat et al. 2013; Bagheri et al. 2013). Previously, the interaction between rice roots and *P. indica* under osmotic stress was not fully explored. We have tried to highlight this interaction and concluded that *P. indica* induces drought tolerance in rice seedlings by improving plant morphological, physiological and molecular aspects.

## Methods

### Growth and cultivation conditions

*P. indica* was grown in Kafer liquid media (Hill and Kafer 2001). The pH of the media was adjusted at 6.5 and temperature at 28 °C. Growing culture was continuously shaken at 130 rpm. Three rice genotypes were used in this study, WC-297 (drought tolerant), Caawa (moderately drought tolerant) and IR-64 (drought susceptible).

The method of Sarma et al. (2011) was used for the effective coating of fungus on rice seeds. Seeds of the genotypes were surface sterilized and coated with a paste of *P. indica* in vermiculite, used as a carrier to adhere spores on seed surface. Seeds without fungus association were treated with only vermiculite. After 10 days of co-cultivation with *P. indica*, seedlings were transferred to a Yoshida nutrition media (Yoshida et al. 1976). After 10 days of acclimatization in this media, a 15% solution of PEG-6000 was added to induce osmotic stress in half of the inoculated and half of the non-inoculated plants. Whereas, 15% PEG 6000 induces − 0.295 MPa of osmotic pressure at 25 °C (Michel and Kaufmann 1973).

### Colonization of fungus: Staining of fungal hyphae and spores

To monitor root colonization, roots were analyzed after 4 days of germination and at the end of the experiment. Roots were thoroughly washed with deionized water, cut into 1 cm length segments which were placed overnight in a 10% KOH solution at room temperature. Roots were then washed 5 times with sterilized H_2_O and incubated in 1% HCl solution for 3 min. Later on, roots were mounted in 0.05% trypan blue in lactophenol for microscopic examination (Michal Johnson et al. 2011).

### Morphological analysis

Root and leaf samples were collected 20 days after the beginning of stress exposure. Under control and osmotic stress the gain in fresh root weight (FRW), fresh shoot weight (FSW), dry root weight (DRW), dry shoot weight (DRW), root length (RL) and shoot length (SL) was determined both from non-inoculated and inoculated plants.

### Elemental analysis of leaves and roots

Root and leaf samples of the rice plants were collected 20 days after the beginning of stress exposure. Samples were dried in a hot air oven, initially for 3 h at 105 °C and then for another 24 h at 80 °C. Approximately 0.1 g of dried leaves and roots was used to determine the concentration of P and Zn. Samples were digested in 5 ml of HNO_3_ at 120 °C for 2 h and then at 80 °C for another 2 h in microwave digester (Microwave 3000; Antoon Paar). After digestion, they were diluted to 20 ml with Mili-Q water, and the elemental contents were determined using an inductively coupled plasma-optical emission spectrometer (ICP-OES;. Optima 8000DV; PerkinElmer).

### Determining the chlorophyll fluorescence

Drought induced change in Fv/Fm was determined 20 days after the beginning of stress exposure, using a fluorometer (IMAG-MAXI, Heinz Walz, Effeltrich, Germany). Plants were placed in the dark for 30 min, then 20 cm long leaves were cut and immediately analyzed.

### Electron microscopy observation of chloroplasts

Chloroplasts in rice leaves were examined 20 days after the beginning of stress exposure, using a transmission electron microscope (Hitachi Model H-7650 TEM). Samples were first fixed in 2.5% glutaraldehyde in phosphate buffer (0.1 M, pH 7.0) for more than 4 h and washed three times with phosphate buffer (0.1 M, pH 7.0) for 15 min. They were fixed with 1% OsO_4_ in phosphate buffer (0.1 M, pH 7.0) for 2 h, and washed three times in phosphate buffer (0.1 M, pH 7.0) for 15 min. They were then dehydrated stepwise using graded ethanol (30%, 50%, 70%, 80%, 90%, 95% and 100%) for 15 min at each step and transferred to absolute acetone for 20 min. Infiltration of the samples was done by placing them in a mixture of absolute acetone and the final spurr resin mixture (1:1) for 1 h at room temperature. Then samples were transferred to a mixture of absolute acetone and final resin mixture (1:3) for 3 h and to final spurr resin mixture for overnight. The samples were finally placed in eppendorf tubes containing spurr resin and heated at 70 °C for more than 9 h. They were sectioned with a LEICA EM UC7 ultratome and sections were stained with uranyl acetate and alkaline lead citrate for 5 and 10 min respectively and then observed in transmission electron microscope (Hitachi Model H-7650).

### Expression pattern of *P5CS* gene

The expression pattern of *P5CS* (proline synthesizing gene) was observed 24 h after the beginning of stress exposure. For primers designing, coding DNA sequences (CDS) were retrieved from The Rice Annotation Project Database (http://rapdb.dna.affrc.go.jp/). Ubiquitin (*UBQ*) was used as a reference gene. Forward and reverse primers of these two genes are given in the Additional file 1: Table S1. TRIzol® reagent (FAVORGEN BIOTECH CORP) was used for the extraction of RNA. Plant leaves were grind in liquid nitrogen and then transferred immediately in microfuge tube holding 700 μl Trizol. This mixture was centrifuged at 12000 rpm for 2 min. Supernatant was shifted in another tube and 300 μl chloroform was added and mixed gently by inversion. The solution was centrifuged to get phase separation at 12000 rpm for 10 min. The upper phase was collected in another microfuge tube, an equal amount of iso-propanol was added followed by an incubation on ice for 10 min. Again centrifuged at 12000 rpm for 10 min, supernatant was discarded and pellet of RNA was collected. Pellet was washed with 75% ethanol. Air dried for 2–3 min and diluted in 25 μl nuclease free water. All centrifugations were done at 4 °C. High quality RNA was used to synthesize cDNA following the protocol of Thermo Fisher Scientific. Gel bands of the PCR products were analyzed with IMAGE. J software. Numerical values showing gel band strength were used to calculate the percentage of change in the expression of *P5CS*.

### Proline analysis

For the determination of proline, the method of Bates et al. (1973) was used. Leaves (0.5 g) collected 20 days after the beginning of stress exposure, were homogenized in 5 mL of 3% aqueous sulphosalicylic acid. The homogenate was filtered through Whatman No.2 filter paper. One milliliter of filtrate was taken and mixed with 1 mL of acid ninhydrin and 1 mL of glacial acetic acid in a test tube. The mixture was briefly vortexed and heated at 100 °C in a water bath for 1 h and terminated the reaction in an ice bath. Four milliliter of toluene were added to the solution and vortexed for 15–20 s. The chromophore containing free proline was aspirated from the aqueous phase in a test tube and warmed to room temperature. The absorbance was measured at 520 nm using a spectrophotometer.

### Estimation of total antioxidant capacity

The method of Prieto et al. (1999) was used for the determination of total antioxidant capacity (TAC)*.* Leaf samples (0.4 g) collected 20 days after the beginning of stress exposure, were homogenized in 50% methanol. The assay mixture containing 900 μl reagent (0.6 M H_2_SO_4_, 28 mM sodium phosphate and 4 mM ammonium molybdate) and 100 μl of enzyme extract was boiled at 95 °C for 30 min. Spectrophotometer absorbance was measured at 695 nm, a higher absorbance (nm) indicating a higher TAC (Prasad et al. 2009).

### Statistical analysis

Experiment was performed by following factorial under completely randomized design. It had three replications (each replication had one plant per pot), three genotypes and four treatments (no osmotic stress, no osmotic stress + *P. indica* inoculation, osmotic stress and osmotic stress + *P. indica* inoculation). Data were analyzed using Analysis of Variance method and least significant difference (LSD_0.05_) (Statistix 8, version 8.1).

## Results

### Establishment of symbiotic association

**Fig. 1:**
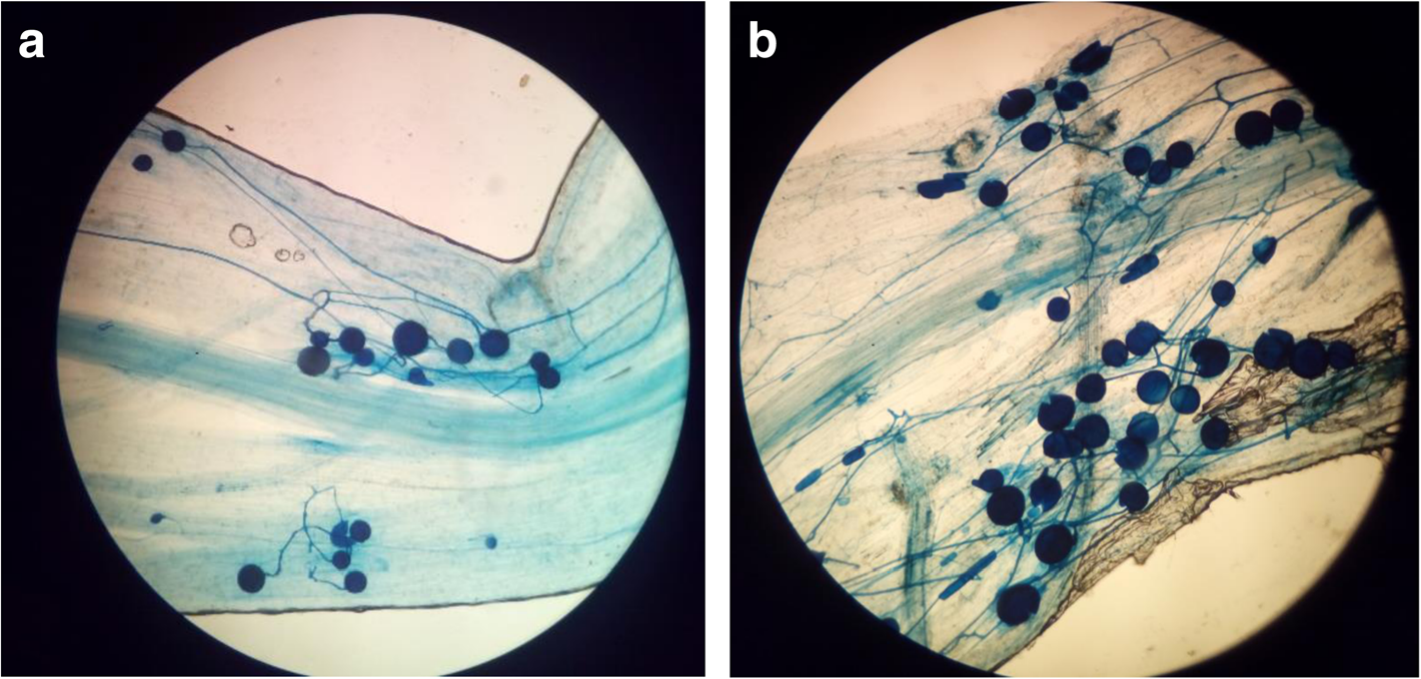
Association of *P. indica* with rice roots. **a** Established symbiosis after 4 days of germination, a network of hyphae and spores is visible within rice roots. **b** Network of hyphae along with attached spores, observed at the end of experiment

*P. indica* successfully colonized rice roots. A strong association of root and fungus was observed after 4 days of germination. Fungus was found to develop spores and hyphae within emerging roots (Fig. 1a). This association was maintained until the end of experiment (Fig. 1b).

### Morphological analysis

**Fig. 2:**
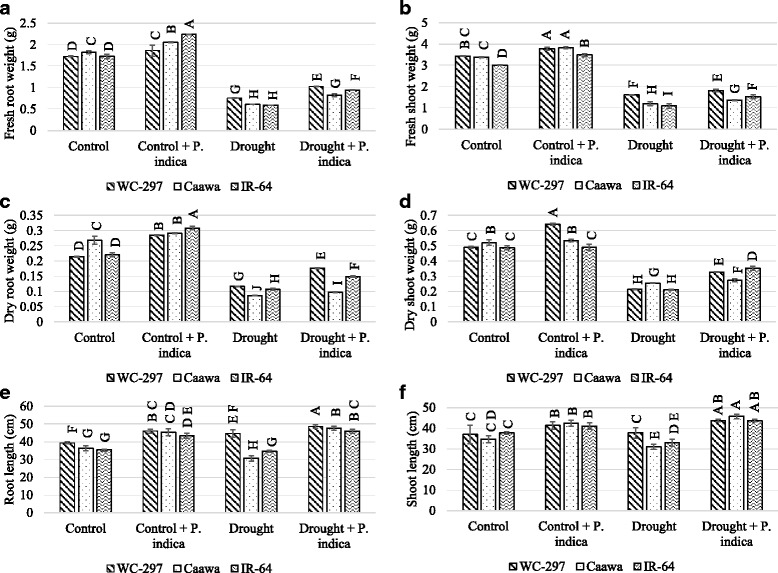
Gain in biomass and length of root and shoot of rice seedlings. The inoculation with *P. indica* increased the plant biomass and the length of root and shoot. **a** Gain in fresh root weight. **b** fresh shoot weight **c** dry root weight **d** dry shoot weight **e** root length and **f** shoot length. Alphabetic on the top of each bar are showing the LSD_0.05_ difference

Seedling biomass and length of root and shoot were significantly improved in inoculated plants (*p* ≤ 0.01, Table 1). In the absence of osmotic stress, the average FRW of the non-inoculated plants of WC-297, Caawa and IR-64 was 1.73, 1.82 and 1.72 g, respectively. When plants were inoculated with *P. indica* the average FRW was increased to 1.86, 2.06 and 2.24 g in the three genotypes, respectively. Under osmotic stress average FRW in WC-297, Caawa and IR-64 was 0.75, 0.61 and 0.59 g in non-inoculated plants and 1.02, 0.82 and 0.94 g in inoculated plants (Fig. 2a). In the absence of osmotic stress, the average FSW of non-inoculated plants were 3.44, 3.38 and 3.01 g in WC-297, Caawa and IR-64, respectively. In inoculated plants it was 3.79, 3.82 and 3.49 g in WC-297, Caawa and IR-64, respectively. Under osmotic stress the average FSW for the non-inoculated plants of these three genotypes was 1.61, 1.20 and 1.09 g and in inoculated plants it was 1.82, 1.37 and 1.53 g in WC-297, Caawa and IR-64, respectively (Fig. 2b). In the absence of osmotic stress, the average DRW of the non-inoculated plants of WC-297, Caawa and IR-64 was 0.21, 0.26 and 0.22 g, respectively. When plants were inoculated with *P. indica* the average DRW was increased to 0.28, 0.29 and 0.31 g in the three genotypes, respectively. Under osmotic stress average DRW in WC-297, Caawa and IR-64 was 0.12, 0.09 and 0.11 g in non-inoculated plants and 0.18, 0.10 and 0.15 g in inoculated plants, respectively (Fig. 2c). In the absence of osmotic stress, the average DSW of non-inoculated plants were 0.49, 0.52 and 0.49 g in WC-297, Caawa and IR-64, respectively. In inoculated plants it was 0.64, 0.53 and 0.49 g in WC-297, Caawa and IR-64, respectively. Under osmotic stress the DSW for the non-inoculated plants of these three genotypes was 0.21, 0.25 and 0.21 g and in inoculated plants it was 0.33, 0.27 and 0.35 g in WC-297, Caawa and IR-64, respectively (Fig. 2d). In the absence of osmotic stress, the average RL of the non-inoculated plants of WC-297, Caawa and IR-64 was 39.33, 36.33 and 35.34 cm, respectively. When plants were inoculated with *P. indica* the average RL was increased to 46.0, 45.0 and 43.0 cm in the three genotypes, respectively. Under osmotic stress average RL in WC-297, Caawa and IR-64 was 44.6, 30.7 and 34.6 cm in non-inoculated plants and 48.0, 47.6 and 46.0 cm in inoculated plants, respectively (Fig. 2e). In the absence of osmotic stress, the average SL of non-inoculated plants were 37.0, 34.6 and 37.7 cm in WC-297, Caawa and IR-64, respectively. In inoculated plants it was 41.3, 42.3 and 41.0 cm in WC-297, Caawa and IR-64, respectively. Under osmotic stress the average SL for the non-inoculated plants of these three genotypes was 37.6, 31.0 and 33.0 cm and in inoculated plants it was 43.6, 45.0 and 43.0 cm in WC-297, Caawa and IR-64, respectively (Fig. 2f).

**Table 1:** Mean squares of absolute values for various seedling traits of the three rice genotypes grown in control, control + *P. indica*, drought and drought + *P. indica*

Residual	Genotype × Treatment	Treatment	Genotype	Source of variation
24	6	3	2	df
0.0018	0.055^**^	3.9^**^	0.0066^**^	Fresh root weight
0.0035	0.067^**^	13^**^	0.45^*^	Fresh shoot weight
0.000024	0.0027^**^	0.068^**^	0.00051^**^	Dry root weight
0.00011	0.0076^**^	0.21^**^	0.0035^**^	Dry shoot weight
1.7	17^**^	306^**^	61^**^	Root length
3.7	14^*^	202^**^	7.2^NS^	Shoot length
0.0000073	0.00066^**^	0.0050^**^	0.0070^**^	Fv/Fm
5.7	229^**^	1333^**^	1088^**^	Proline
0.00045	0.0056^**^	0.042^**^	0.0085^**^	Total antioxidant capacity
0.0086	0.68^**^	22^**^	0.015^NS^	Phosphorus in root
0.018	0.056^*^	20^**^	0.14^**^	Phosphorus in shoot
0.0000012	0.000058^**^	0.00046^**^	0.000034^**^	Zinc in root
0.0000029	0.00018^**^	0.0020^**^	0.0000087^NS^	Zinc in shoot

*, ** and NS indicates significant differences at *p* ≤ 0.05, *p* ≤ 0.01 and non-significant (*p* > 0.05), respectively

### Elemental analysis

**Fig. 3:**
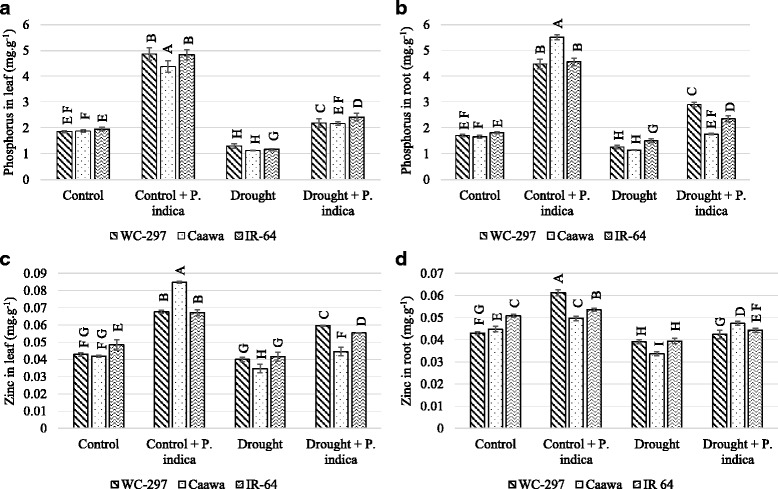
Phosphorus and zinc concentration in rice leaf and root. The inoculation with *P. indica* increased the level of P and Zn in leaf and root. **a** Increase in the concentration of P in leaves. **b** P concentration in roots. **c** Zn concentration in leaves. **d** Zn concentration in roots. Alphabetic on the top of each bar are showing the LSD_0.05_ difference

The concentration of P and Zn were significantly improved in the presence of root symbiosis (*p* ≤ 0.01, Table 1).The uptake of P was almost double in inoculated than in non-inoculated plants. In the absence of osmotic stress, average P concentration in leaves of the non-inoculated plants of WC-297, Caawa and IR-64 was 1.85, 1.88 and 1.95 mg.g^− 1^, respectively. When plants were inoculated with *P. indica*, P concentration increased to 4.86, 4.38 and 4.84 mg.g^− 1^ in the three genotypes, respectively. Under osmotic stress, P leaf concentration in WC-297, Caawa and IR-64 was 1.3, 1.13 and 1.16 mg.g^− 1^ in non-inoculated plants, and 2.19, 2.16 and 2.42 mg.g^− 1^ in inoculated plants (Fig. 3a). Almost similar change in P level was noticed when roots were analyzed. In the absence of osmotic stress, P concentration in roots of non-inoculated plants were 1.71, 1.65 and 1.81 mg.g^− 1^ in WC-297, Caawa and IR-64, respectively. In inoculated plants, P concentration increased to 4.48, 5.51 and 4.56 mg.g^− 1^ in WC-297, Caawa and IR-64, respectively. Under osmotic stress P concentration of the three genotypes was 1.26, 1.14 and 1.49 mg.g^− 1^ in non-inoculated plants and 2.90, 1.76 and 2.35 mg.g^− 1^ in inoculated plants in WC-297, Caawa and IR-64, respectively (Fig. 3b).

The symbiotic association of the rice plant with *P. indica* also increased leaves and roots Zn concentration. In the absence of osmotic stress, leaves Zn concentration of non-inoculated plants of WC-297, Caawa and IR-64 was 0.042, 0.041 and 0.048 mg.g^− 1^, respectively. In inoculated plants, Zn concentration increased to 0.068, 0.084 and 0.067 mg.g^− 1^ in the three genotypes, respectively. Under osmotic stress, the average leaf Zn concentration of non-inoculated plants of WC-297, Caawa and IR-64 were 0.040, 0.034 and 0.041 mg.g^− 1^, respectively. In inoculated plants, leaf Zn concentration increased to 0.06, 0.045 and 0.055 mg.g^− 1^ in the three genotypes, respectively (Fig. 3c). In the absence of osmotic stress, root Zn concentration was 0.043, 0.045 and 0.051 mg.g^− 1^, in non-inoculated plants and 0.061, 0.05 and 0.054 mg.g^− 1^ in inoculated plants of WC-297, Caawa and IR-64, respectively. Under osmotic stress, root Zn concentration was 0.039, 0.034 and 0.039 mg.g^− 1^ in non-inoculated plants and 0.042, 0.047 and 0.044 mg.g^− 1^ in inoculated plants of WC-297, Caawa and IR-64, respectively (Fig. 3d).

### Expression pattern of *P5CS* gene

**Fig. 4:**
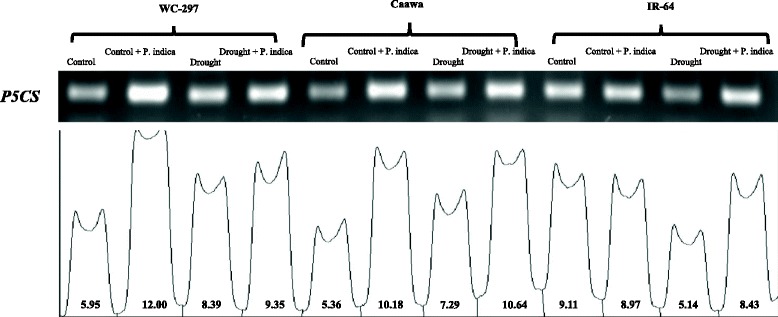
Change in the expression pattern of *P5CS*. *P. indica* increased the expression of *P5CS* both in the presence and absence of osmotic stress. With a little exception for IR-64, where without inoculation, a little decrease in expression was recorded under osmotic stress

The expression of *P5CS* gene was influenced by the presence of *P. indica*. The change in the expression of *P5CS* was obvious both in the presence and absence of osmotic stress (Fig. 4). In the absence of osmotic stress, the inoculation by *P. indica* increased the expression of this gene up to 102 and 90% in WC-297 and Caawa, respectively. In IR-64 a decrease of 1.5% was noted in the expression of *P5CS*. Under osmotic stress, symbiotic association also increased the expression of *P5CS* gene by 11, 46 and 64% in WC-297, Caawa and IR-64, respectively. Osmotic stress induced an increase of the expression of *P5CS* in non-inoculated plants of WC-297 and Caawa (41% and 36%, respectively) and decrease of this expression (43%) in IR-64.

### Proline concentration and total antioxidant capacity

**Fig. 5:**
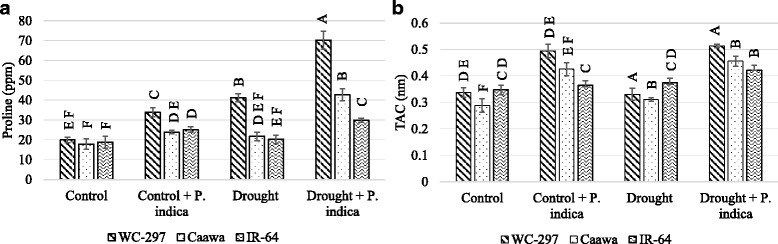
Change in the concentration of proline and TAC. The inoculation with *P. indica* increased the concentration of proline and total antioxidant capacity (TAC) in both the absence and presence of osmotic stress. **a** Increase in proline concentration. **b** Increase in TAC. Alphabetic on the top of each bar are showing the LSD_0.05_ difference

The concentration of proline and the level of TAC were significantly improved in the presence of root symbiosis (*p* ≤ 0.01, Table 1). Osmotic stress increased the level of both proline and TAC in rice leaves, while the inoculation of the plant with *P. indica* boosted the concentration of these beneficial compounds. In the absence of osmotic stress, proline concentration in non-inoculated plants was 19.96, 17.92 and 18.83 ppm in WC-297, Caawa and IR-64, respectively. In inoculated plants, proline concentration was increased to 33.94, 23.94 and 25.18 ppm. Under osmotic stress proline concentration was 41.31, 21.74 and 20.23 ppm in non-inoculated plants and 70.23, 42.81 and 29.96 ppm in inoculated plants, in WC-297, Caawa and IR-64, respectively (Fig. 5a). Plant analysis for TAC also shown similar results. In the absence of osmotic stress, TAC in non-inoculated plants was 0.34, 0.29 and 0.35 nm in WC-297, Caawa and IR-64, respectively. In inoculated plants TAC was 0.49, 0.43 and 0.36 nm in WC-297, Caawa and IR-64, respectively. In the presence of osmotic stress TAC was 0.33, 0.31 and 0.37 nm in non-inoculated plants and 0.51, 0.46 and 0.42 nm in inoculated plants, in WC-297, Caawa and IR-64, respectively (Fig. 5b).

### Chlorophyll fluorescence (Fv/Fm)

**Fig. 6:**
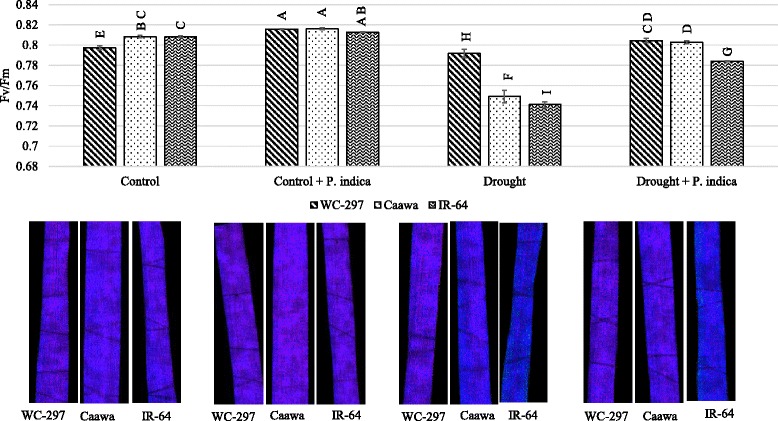
Change in Fv/Fm. The inoculation with *P. indica* increased Fv/Fm in both the absence and presence of osmotic stress. Under each bar of the graph there is a picture of the leaf section showing Fv/Fm activity. Alphabetic on the top of each bar are showing the LSD_0.05_ difference

Fv/Fm was significantly improved in the presence of root symbiosis (*p* ≤ 0.01, Table 1). Water limited environment affected the photosynthetic machinery and so, reduced Fv/Fm. This decrease was higher in IR-64 than in Caawa and WC-297. The symbiotic association increased Fv/Fm both in the presence and absence of osmotic stress. In the absence of osmotic stress, the value of Fv/Fm in non-inoculated plants of WC-297, Caawa and IR-64 was 0.79, 0.81 and 0.81, respectively. In inoculated plants, it was 0.82, 0.82 and 0.81 in the three genotypes. In the presence of osmotic stress, Fv/Fm was 0.79, 0.75 and 0.74 in non-inoculated plants and 0.80, 0.80 and 0.78 in inoculated plants, in WC-297, Caawa and IR-64, respectively (Fig. 6).

### Electron microscopy

**Fig. 7:**
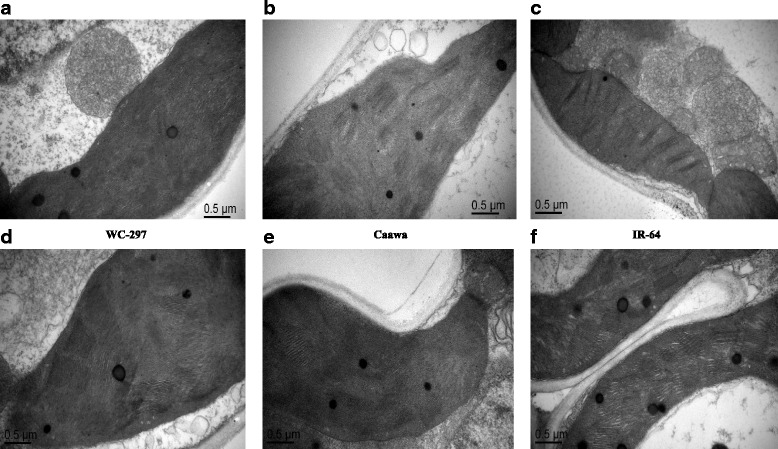
Chloroplast of rice genotypes in the absence of osmotic stress. **a-c** Chloroplasts of the non-inoculated plants of WC-297, Caawa and IR-64, respectively. **d-f** Chloroplasts of the inoculated plants of WC-297, Caawa and IR-64, respectively. Grana in chloroplasts are better organized in rice plants inoculated with *P. indica*

**Fig. 8:**
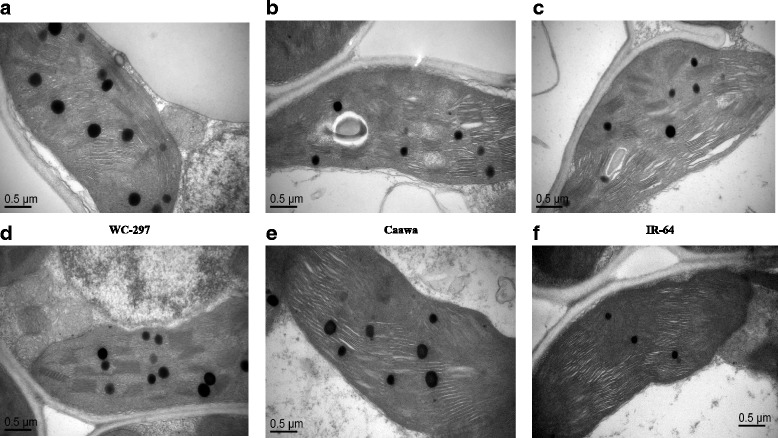
Chloroplast of rice genotypes in the presence of osmotic stress. **a-c** Chloroplasts of the non-inoculated plants of WC-297, Caawa and IR-64, respectively. **d-f** Chloroplasts of the inoculated plants of WC-297, Caawa and IR-64, respectively. Grana in chloroplasts are better organized in rice plants inoculated with *P. indica*

The inoculation with *P. indica* also stabilized the stacking of grana in chloroplasts in both the absence and presence of osmotic stress. In the absence of osmotic stress the grana were more organized when plants were inoculated with *P. indica* (Fig. 7). Similarly, under osmotic stress the grans were healthy when plants were inoculated with *P. indica* (Fig. 8). In non-inoculated plants, under osmotic stress, the highest damage to chloroplasts was noted in IR-64. Some damage was also observed in Caawa. In WC-297 the chloroplasts were healthy even under drought stress. In inoculated plants, the grana in chloroplast were well organized under osmotic stress.

## Discussion

The effects of osmotic stress have been frequently reported in rice. Osmotic stress hinders mineral uptake, transport and distribution (Zain et al. 2014), reduces chlorophyll content, disintegrate the grana in chloroplasts and disturbs photosynthetic efficiency (Farooq et al. 2009b; Zain et al. 2014; Swapna and Shylaraj 2017; Korres et al. 2017). Several research papers has been published during the last two decades which emphasize the supportive role of *P. indica* in mitigating the osmotic stress in different host plants. It has been reported that *P. indica* serves as a bioregulator, biofertilizer and bioprotector for many host plants. Regardless of the stress, *P. indica* has evolved to protect its food source-the plant. It activates different signaling, transport, metabolic and developmental programs in plants (Ngwene et al. 2016; Gill et al. 2016; Bakshi et al. 2017). We demonstrate that *P. indica* increased the rice seedling biomass, length of root and shoot, P and Zn concentration in root and shoot, proline concentration, TAC, Fv/Fm and the expression of *P5CS*. Most of the previous research articles elaborated the mechanisms that how *P. indica* ameliorates the drastic effects of salt, heavy metal and some biotic stresses in rice plant. It had been reported that this symbiotic fungus increase the concentration of proline, antioxidants and decreases the concentration of malondialdehyde under these stresses (Bagheri et al. 2014; Nassimi and Taheri 2017; Mohd et al. 2017). Most of the yield damage in rice is due to the water shortage. There is very less literature available explaining the rice and *P. indica* symbiosis under osmotic stress. So, there is a need to investigate the role of *P. indica* for inducing drought tolerance in rice. This research article highlighted the *P. indica* induced improvement in some of the important features of rice plants under osmotic stress. We determined that *P. indica* significantly increased the rice plant biomass and the length of root and shoot under control and osmotic stress. Similar growth improvement was reported in Arabidopsis and many other field crops (Franken 2012; Im et al. 2002). In our study, the analysis of mineral profile showed that symbiotic association almost doubled the concentration of both P and Zn in leaves and roots of the rice plant. This increase in mineral acquisition was observed in all three genotypes both in the presence and absence of osmotic stress. The increase in the availability of both macro and micronutrients in the rhizosphere due to the association with *P. indica* has been reported in many crops (Franken 2012). Present study, focused on the uptake of P and Zn as these two elements have been described to have an important role in maintaining plant growth and development under water-limited environments (Ngwene et al. 2016; Tariq et al. 2017). Sufficient supply of P increases drought tolerance by inducing deeper rooting and higher inorganic phosphorus (Pi) supply for carbon assimilation in leaves. It is also responsible for maintaining optimum leaf relative water contents (Tariq et al. 2017). Similarly, Zn is critical for the growth of rice plants under stress environments as it is the part of antioxidative enzymes like SOD and CAT. Proper concentration of Zn in soil helps plant avoiding ROS damage under abiotic stresses (Cakmak 2000). The effect of the inoculation with *P. indica* on P and Zn concentration, observed in this study, has also been reported in other crops by Franken (2012) and Padash et al. (2016).

Fv/Fm is an important marker to determine the effect of stress on the photosystem II in plants (Murchie and Lawson 2013). In present research, *P. indica* increased Fv/Fm both in control and osmotic stressed plants of rice. Previously Sherameti et al. (2008) and Bakshi et al. (2017) reported that *P. indica* increased the level of Fv/Fm in *Arabidopsis thaliana* under abiotic stress. Similarly, it also stabilized the grana in chloroplasts. Under osmotic stress it helped maintaining grana in proper stacked arrangement, thus protecting the photosynthetic system. .

The association with *P. indica* also altered the expression of *P5CS*. *P. indica* increased the expression of this gene under both control and stressed environments. This gene is involved in the synthesis of proline, an amino acid which is critical for plant growth and development. Proline acts as both an osmotic agent and a radical scavenger (Kishor et al. 2014; Kishor et al. 2015). The supportive role of *P. indica* in increasing the synthesis of this protective compound was also evidenced by our experiment. The accumulation of proline due to osmotic stress was more than double in inoculated plants of WC-297 and Caawa and almost double in inoculated plants of IR-64, compared to non-inoculated plants. An increase in TAC was also associated with the presence of the endophyte. This increase in TAC was significant in all three genotypes. Increase in proline and TAC accumulation due to the inoculation with *P. indica* has also been reported in other crops by Waller et al. (2005) and Prasad et al. (2013). Most of the rice inoculation with *P. indica* had been performed to mitigate the salinity, heavy metal and some biotic stresses. We have tested the association of *P. indica* with rice plant under osmotic stress and it was summarized that this fungus improved plant performance under water deficient environment as it is already reported for inducing tolerance in rice under many other stresses.

## Conclusion

*P. indica* is known to help maintaining growth and yield in many field crops under drought stress, but the underlying mechanisms are not fully elucidated. In the present study, *P. indica* was found to improve morphological traits, mineral uptake and protect the photosynthetic machinery in rice plants submitted to osmotic stress. Moreover, the inoculation with *P. indica* increased Fv/Fm, up regulated the expression of *P5CS*, raised the accumulation of proline and TAC. These results confirmed that the endophyte has a significant role in protecting the rice plant against osmotic stress. Further investigation is needed, however, to validate these effects of the inoculation in rice fields under various drought scenarios and use the inoculation with *P. indica* as an additional agronomical tool to improve rice grain yield under drought-prone conditions.

## Additional file

Additional file 1: **Table S1.** Forward and reverse primers of the genes under study. (DOCX 14 kb)

## Abbreviations

Caawa: Moderately drought tolerant genotype
CDS: Coding DNA sequence
Fv/Fm: Maximum quantum yield of PSII
IR-64: Drought susceptible genotypes
P: Phosphorus
*P. indica*: *Piriformospora indica*
P5CS: Pyrroline-5-carboxylate synthase
PEG-6000: Polyethylene glycol-6000
ROS: Reactive oxygen species
TAC: Total antioxidant capacity
TEM: Transmission electron microscope
WC-297: Drought tolerant genotypes
Zn: Zinc
