# Supplementary material for: Inoculation with the endophyte Piriformospora indica significantly affects mechanisms involved in osmotic stress in rice
Source: Rice (N Y). 2018 May 24;11:34. doi: 10.1186/s12284-018-0226-1 (PMC5968016; doi:10.1186/s12284-018-0226-1)
Supplement: Supplementary file 1 — Table S1. Forward and reverse primers of the genes under study. (DOCX 14 kb) [file 12284_2018_226_MOESM1_ESM.docx]

Table S1. Forward and reverse primers of the genes under study.

| **Gene Name** | **Gene ID** | **Forward primer** | **Reverse primer** |
| --- | --- | --- | --- |
| P5CS | >Os05t0455500-01 | TTGTGCCGCTGTTGGTCAGA | AGTGAGTTGCTCCCGGAACTT |
| UBQ | >Os01t0125900-01 | ATGGAACTGCCAGTGATGGT | AGCCGAGTCACAAAGCCAAA |
